# Caveolin-1 in Kidney Chronic Antibody-Mediated Rejection: An Integrated Immunohistochemical and Transcriptomic Analysis Based on the Banff Human Organ Transplant (B-HOT) Gene Panel

**DOI:** 10.3390/biomedicines9101318

**Published:** 2021-09-26

**Authors:** Alessandro Gambella, Antonella Barreca, Simona Osella-Abate, Emanuel Bottasso, Manuela Maria Giarin, Mauro Papotti, Luigi Biancone, Jasna Metovic, Giammarco Collemi, Paola Cassoni, Luca Bertero

**Affiliations:** 1 Pathology Unit, Department of Medical Sciences, University of Turin, Via Santena 7, 10126 Turin, Italy; alessandro.gambella@unito.it (A.G.); giammarco.collemi@gmail.com (G.C.); luca.bertero@unito.it (L.B.); 2 Pathology Unit, “Città della Salute e della Scienza di Torino” University Hospital, Via Santena 7, 10126 Turin, Italy; abarreca@cittadellasalute.to.it (A.B.); emanuel.bottasso@unito.it (E.B.); mgiarin@cittadellasalute.to.it (M.M.G.); 3 Molecular Pathology Unit, “Città della Salute e della Scienza di Torino” University Hospital, Via Santena 5, 10126 Turin, Italy; sosellaabate@cittadellasalute.to.it; 4 Pathology Unit, Department of Oncology, University of Turin, Via Santena 7, 10126 Torino, Italy; mauro.papotti@unito.it (M.P.); jasna.metovic@unito.it (J.M.); 5 Renal Transplantation Center ‘A. Vercellone’, Division of Nephrology Dialysis and Transplantation, Department of Medical Sciences, Città della Salute e della Scienza Hospital and University of Turin, 10124 Torino, Italy; luigi.biancone@unito.it

**Keywords:** caveolin-1, immunohistochemistry, B-HOT, chronic antibody-mediated rejection, C4d, kidney transplantation

## Abstract

Caveolin-1 overexpression has previously been reported as a marker of endothelial injury in kidney chronic antibody-mediated rejection (c-ABMR), but conclusive evidence supporting its use for daily diagnostic practice is missing. This study aims to evaluate if Caveolin-1 can be considered an immunohistochemical surrogate marker of c-ABMR. Caveolin-1 expression was analyzed in a selected series of 22 c-ABMR samples and 11 controls. Caveolin-1 immunohistochemistry proved positive in peritubular and glomerular capillaries of c-ABMR specimens, irrespective of C4d status whereas all controls were negative. Multiplex gene expression profiling in c-ABMR cases confirmed Caveolin-1 overexpression and identified additional genes (*n* = 220) and pathways, including MHC Class II antigen presentation and Type II interferon signaling. No differences in terms of gene expression (including Caveolin-1 gene) were observed according to C4d status. Conversely, immune cell signatures showed a NK-cell prevalence in C4d-negative samples compared with a B-cell predominance in C4d-positive cases, a finding confirmed by immunohistochemical assessment. Finally, differentially expressed genes were observed between c-ABMR and controls in pathways associated with Caveolin-1 functions (angiogenesis, cell metabolism and cell–ECM interaction). Based on our findings, Caveolin-1 resulted as a key player in c-ABMR, supporting its role as a marker of this condition irrespective of C4d status.

## 1. Introduction

The main goal of kidney transplantation is to improve the quality of life of patients with end-stage kidney disease, avoiding renal replacement therapy. Over the last 40 years, many advances have been made to preserve and extend allograft survival; however, despite these efforts, graft failure still occurs, and about 10–15% of cases require a second transplantation [1]. The main cause of late kidney transplant failure is chronic antibody-mediated rejection (c-ABMR), clinically characterized by a progressive worsening of graft function with an increment of creatinine plasma levels and a variable degree of proteinuria [2,3,4,5]. Although kidney biopsy represents the gold standard diagnostic procedure of c-ABMR, early morphologic signs detectable by light microscopy are limited. Current Banff diagnostic criteria of c-ABMR include the presence of transplant glomerulopathy (TG), peritubular capillary basement membrane multilayering and/or transplant arteriopathy, which can be promptly identified in advanced stages only once the potential efficacy of treatments is reduced. Conversely, early changes can be demonstrated by transmission electron microscopy only, a tool with restricted availability [6,7,8,9,10]. A significant number of c-ABMR cases (i.e., chronic active ABMR) also display signs of activity (e.g., glomerulitis and/or peritubular capillaritis) and linear C4d staining in peritubular capillaries (PTC) [6].

C4d immunohistochemical staining (IHC) represents a compelling diagnostic aid for c-ABMR, and its introduction was a striking development in humoral-related pathology diagnostics [11,12,13,14,15]. However, a considerable number of antibody-mediated rejection cases turn out to be C4d negative, hence a specific C4d-negative category was introduced in the 2013 Banff classification [16,17,18,19,20,21]. C4d-negativity may be explained by fluctuating donor specific antibodies (DSA) levels, resulting in little or absent complement activation at the time of biopsy, or by activation of C4d-independent mechanisms leading to antibody-mediated tissue injury [22]. Indeed, antibody-dependent cellular cytotoxicity is a well-characterized humoral response effector mechanism which is thought to play a crucial role in C4d-negative cases, mostly by recruitment of NK cells [23]. Conversely, C4d expression is also observed in cases with no clinical suspect nor histological proof of antibody-mediated rejection, particularly in cases of ABO-incompatible transplantation [24,25,26].

Recently, molecular profiling has been proposed to overcome these diagnostic challenges. In 2020, the Banff Molecular Diagnostics Working Group proposed the Banff Human Organ Transplant (B-HOT) transcriptomic panel to reliably and reproducibly evaluate transplant-related pathological conditions [27]. Based on extensive multiplex analysis, this panel was customized to characterize messenger RNA expression of 758 genes related to transplant rejection and is nowadays available through NanoString Technologies as the nCounter^®^ Human Organ Transplant Panel [27]. Although molecular profiling represents an intriguing and promising strategy, it is not widely and routinely available because of the required resources, facilities, and expertise. Thus, reliable, swift, and cost-effective surrogates such as IHC markers are warranted and probably represent the optimal solution for daily diagnostic practice.

In this regard, Caveolin-1 (Cav-1), has been demonstrated to be overexpressed in antibody-mediated rejection by injured vascular endothelial cells [28,29,30,31,32,33,34,35] and was therefore included as a potential molecular marker of this condition in the 2017 Banff classification [36]. However, a proper orthogonal validation of Cav-1 diagnostic significance in c-ABMR is still missing.

As such, we set out to fully characterize and confirm the role of Cav-1 in kidney c-ABMR. A series of c-ABMR samples with both inactive and active features were selected from our records, aiming to:

1 Establish Cav-1 as a reliable IHC marker of c-ABMR, irrespectively of C4d status, through transcriptomic profiling.
2 Assess the molecular profile of c-ABMR cases and improve its transcriptomic characterization using the B-HOT-derived nCounter^®^ Human Organ Transplant Panel.
3 Investigate genes related to Cav-1 expression to elucidate its role in c-ABMR.

## 2. Materials and Methods

### 2.1. Case Selection and Database Construction

This is a retrospective single-center study analyzing a series of kidney allograft biopsies with a confirmed diagnosis of c-ABMR. Twenty-two cases diagnosed as c-ABMR by indication biopsy between August 2014 and April 2020 were retrieved from the Pathology Unit records of the Città della Salute e della Scienza Hospital of Torino. Cases were selected according to the latest Banff meeting report, thus presenting consistent histopathological features (e.g., TG) and C4d expression together with DSA-positivity, and collected to include both C4d-positive and C4d-negative cases. To avoid potential confounders, samples with other concurrent pathologies, related or not to transplantation, were excluded. Eleven biopsies of allograft kidneys with complete clinical/follow-up data, adequate biopsy material, and no laboratory, clinical, nor histopathological evidence of rejection were also retrieved as a control group. Demographic, clinical, and pathological data were collected from patients’ clinical files/original diagnostic reports and entered in a pseudonymized database.

Samples had been collected by incisional transcutaneous biopsies, processed, and stained according to our laboratory protocols (Methods M1). According to the most recent Banff classification, additional pathological features related to c-ABMR [i.e., focal segmental glomerulosclerosis (FSGS), glomerulitis, peritubular capillaritis, and TG] and C4d IHC staining were evaluated and graded. Cases were then independently reviewed by three pathologists (A.G., A.B., E.B.), and, in case of disagreement, the findings were discussed collectively to reach a consensus classification.

**Figure 1:**
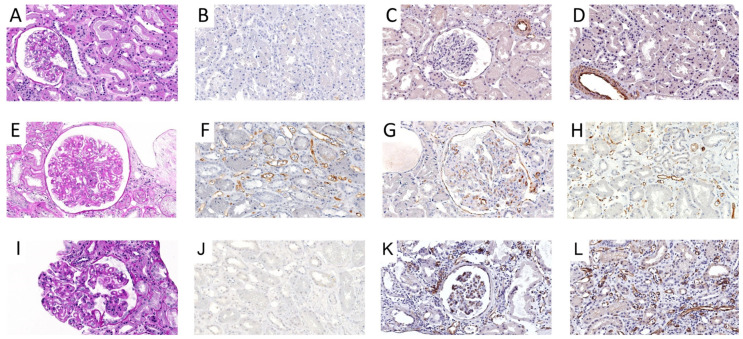
The control group (**A**–**D**) showed no morphological signs of rejection (**A**) and resulted negative to C4d (**B**) and Cav-1 IHC, the latter both in glomerular (**C**) and peritubular (**D**) capillaries. In Cav-1 IHC negative cases (Grade 0), arterial vessels were evaluated as on-slide positive controls (**C**,**D**). The c-ABMR group was composed of C4d positive (**E**–**H**) and C4d negative (**I**–**L**) cases, both presenting features of TG (**E**,**I**). The former group presented a positive C4d IHC in peritubular capillaries (**F**) and was then scored accordingly to the Banff classification. Accordingly, Cav-1 IHC showed a diffuse and intense positivity in glomerular (**G**) and peritubular (**H**) capillaries. Although showing TG features (**I**), C4d negative cases presented no expression of C4d (**J**). Conversely, Cav-1 maintained the positivity both in glomerular (**K**), where Cav-1 perfectly traced remodeled segments of basement membrane, and peritubular (**L**) capillaries, thus supporting the diagnosis of c-ABMR. Original magnification 300×.

Cav-1 IHC staining was performed using the N-20 clone (Santa Cruz Biotechnology, Santa Cruz, CA, USA). Since no grading score had been defined so far, we built a C4d-analogue grading system. Cav-1 was scored both in peritubular and glomerular capillaries (Figure 1 and Table 1). 

To evaluate the inflammatory response phenotype, IHC stain for CD79alfa, CD3, and CD56 were performed using the JCB117, LN10, and CD564 clones (Leica Biosystems, Buffalo Grove, IL, USA), respectively. The inflammatory population was then assessed and compared by evaluating inflammatory response per high-power field. In particular, biopsies were first evaluated with hematoxylin and eosin to identify the most representative hot spot of inflammation. Once selected, we counted the number of B (CD79alfa-positive), T (CD3-positive), and NK (CD56-positive) cells within each hot spot.

In addition, Cav-1 IHC staining was also performed and graded in an additional exploratory series of transplant kidney diseases (enlisted in Table S1)

### 2.2. NanoString^®^ Gene-Expression Profiling

Up to four ten-μm-thick sections were obtained from each alcohol-formalin-acetic acid (AFA) fixed-paraffin embedded tissue-block to collect the 300 ng of RNA required for analyses. RNA was isolated and extracted using the tissue RNA Isolation Kit (Roche Diagnostics GmbH, Mannheim, Germany), and then concentration was assessed with the NanoDrop spectrophotometer (Thermo Fisher Scientific Inc., Waltham, MA, USA). The samples with a low RNA input (<20 ng/uL) were concentrated with Eppendorf^®^ Concentrator Plus (Eppendorf AG, Hamburg, Germany). Total RNA from each sample was then hybridized to the nCounter^®^ Human Organ Transplant Panel (NanoString Technologies, Seattle, WA, USA). This panel evaluates mRNA expression of 758 target genes and 12 internal reference genes for data normalization. Each assay also includes a Panel Standard: a pool of synthetic DNA oligonucleotides corresponding to the target sequence of each of the 770 unique probe targets allowing normalization of user, instrument, and lot-to-lot variations.

Expression data were normalized and analyzed with the nSolver Analysis Software (version 4.0.70). Background correction was applied subtracting the mean count of negative controls plus one standard deviation. The means of the supplied controls and of the housekeeping genes were used to normalize the measured expression values. Additionally, the Advanced Analysis module (version 2.0.115) was used to perform differential expression analyses.

### 2.3. Statistical Analysis

Statistical analyses were performed using Stata/MP 15.0 Statistical Software (StataCorp, College Station, TX, USA) using a <0.05 significance level for two-tailed tests. For categorical variables, frequencies were provided, and characteristics were compared using the Chi-square test with Bonferroni corrections. Continuous variables were summarized as median and intervals, performing the *T*-test or ANOVA test with Bonferroni corrections for multiple comparisons. The reverse Kaplan–Meier method was applied to calculate outcome times which were summarized as median values and interquartile ranges (IQR) and compared with the log-rank test.

## 3. Results

### 3.1. Clinical and Histopathological Data

c-ABMR samples were mainly from male patients (15/22) with a median age at diagnosis of 54 years. Native kidney diseases were mostly represented by idiopathic chronic kidney disease (5/22) and immune-mediated glomerulonephritis (5/22). All cases received a single kidney transplant (22/22), mostly from a deceased donor (17/22). They all tested positive for DSA and mainly presented class II DSA (12/22). In our series, ten cases presented signs of FSGS. Histological features of active microvascular inflammation were observed in most cases (14/22), and ten of them had features consistent with both glomerulitis and peritubular capillaritis, the former with g 1 and the latter with ptc 2 as the most represented scores (6/11 and 10/13, respectively). All cases presented TG, mostly scored 2–3 (19/22 cases), with substantially negative immunofluorescence or unspecific mild granular IgM and complement fragments positivity thus excluding immune-mediated glomerulonephritis. c-ABMR patients had a median rejection time of 5.6 years (IQR: 3.6–9.8) and a median follow-up time of 9.9 years (IQR: 6.4–13.3). Proteinuria (PTO) and creatininemia (Crs) values were the only clinicopathological variables statistically related with c-ABMR (*p* = 0.026 and *p* = 0.013). The control samples were mainly females (6/11) with a median age of 49 years, mostly collected as protocol biopsies (8/11). They showed normal histological features (9/11) or alterations consistent with acute calcineurin inhibitor nephrotoxicity (2/11), and unspecific arterial intimal fibrosis (5/11) or arteriolar hyalinosis (7/11). None showed features related to rejection or tested positive for DSA. Data are summarized in Table 2 and Figure S1.

### 3.2. C4d Immunohistochemical Assessment

C4d IHC expression was evaluated in peritubular capillaries and resulted negative (C4d0) in twelve cases (12/22), while most of the C4d-positive cases (6/10) showed diffuse and intense staining (C4d3). C4d negative cases showed a median rejection time of 4.3 years (IQR: 2.2–6.1), that was significantly shorter (*p* = 0.023) than C4d positive cases (median rejection time of 8.5 years; IQR: 5.6–20.4). Conversely, the median follow-up time was not significantly different (*p* = 0.061) comparing C4d negative (median follow-up time of 7.4 years; IQR: 5.6–12.6) and C4d positive (median follow-up time of 10.4 years; IQR: 8.7–21.3) cases. The relationship between the clinical and histopathological variables and C4d status is summarized in Table 3 and Figure S2. All control cases were negative for C4d (C4d0).

### 3.3. Cav-1 Immunohistochemical Assessment

Cav-1 was positive in all c-ABMR cases (22/22), while no control case (0/11) expressed it (*p* < 0.0001). According to our scoring system, the most represented score in peritubular capillaries was Grade III (20/22), while in glomeruli was Grade II (11/22) (Table 4).

Cav-1 positivity in glomerular capillaries perfectly recapitulated the remodeled segments of basement membrane (Figure 1). Considering the c-ABMR population only and stratifying according to C4d expression, the most represented score for peritubular capillaries was Grade III for both C4d negative (10/12) and C4d positive cases (10/10), while glomerular capillaries expression was mainly scored as Grade II in C4d negatives (8/12) and Grade III in C4d positives (6/10). Cav-1 expression was not correlated with C4d positive or negative status, either evaluating peritubular or glomerular capillaries expression (Table 5).

To support the reliability of Cav-1 in the setting of c-ABMR, we evaluated its IHC expression in an exploratory series of additional transplant kidney diseases. In particular, we observed that Cav-1 was completely negative (Grade 0) in both glomerular and peritubular capillaries in cases of arteriosclerosis-related vascular injury (2 cases), renal interstitial fibrosis (1 case), post-transplant membranous glomerulonephritis (2 cases), and acute pyelonephritis (1 case) (Figure S3).

Cases of acute tubular necrosis (1 case), diabetic nephropathy plus recurrent 2,8 dihydroxyadenine (2,8 DHA) nephropathy (1 case), and T-Cell Mediated Rejection (TCMR) (5 cases) presented a minimal Cav-1 expression in isolated sparse peritubular capillaries (Grade I) only, while glomerular capillaries were negative (Grade 0) in all cases (Figure S3).

In addition, we evaluated Cav-1 expression in cases of antibody-mediated rejection with other superimposed conditions. We assessed Cav-1 expression in active antibody-mediated rejection (10 cases), mixed c-ABMR and TCMR (1 case), and mixed c-ABMR (confirmed by electron microscopy) and IgA nephropathy (4 cases). In all these conditions, Cav-1 resulted strongly and diffusely expressed in glomerular and peritubular capillaries (Figure S3). Cav-1 scores of the above entities are detailed in Table S1.

Although based on a small group of cases for each condition, this pilot IHC analysis confirmed the reliability of focal and diffuse Cav-1 positivity (Grade II-III) to specifically detect antibody-mediated rejection injury in a wide range of kidney diseases.

### 3.4. Gene Expression Profiling: c-ABMR versus Control Group

**Figure 2:**
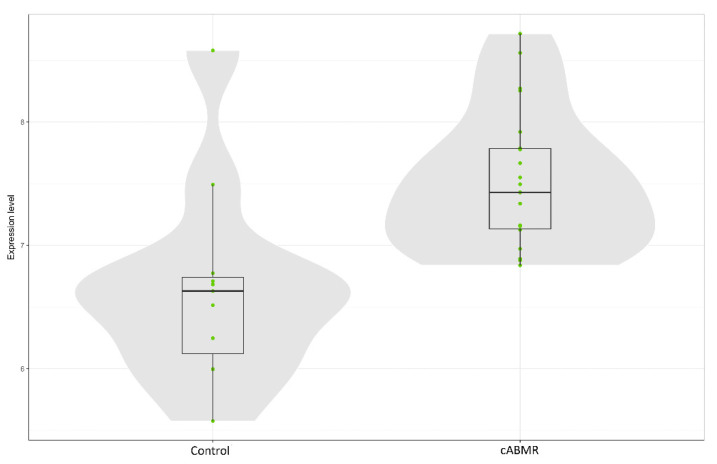
Cav-1 gene expression. The box plot represents the distribution of Cav-1 expression comparing c-ABMR and the control group. The grey areas represent the estimated distribution of Cav-1 expression while the green dots represent each sample Cav-1 log2 expression.

Gene expression analysis was performed in all 33 samples; quantity and quality of extracted mRNA were adequate in all cases. Comparing c-ABMR cases with the control group, up to 221 genes (representing 37 of the 38 annotated pathways examined by the panel) resulted significantly more expressed in c-ABMR (one-hundred-seventy genes with a *p*-value < 0.01 and fifty-one with a *p*-value < 0.05, respectively). Cav-1 was one of the genes significantly more expressed in c-ABMR compared with the control group (*p* < 0.05) (Figure 2). 

**Figure 3:**
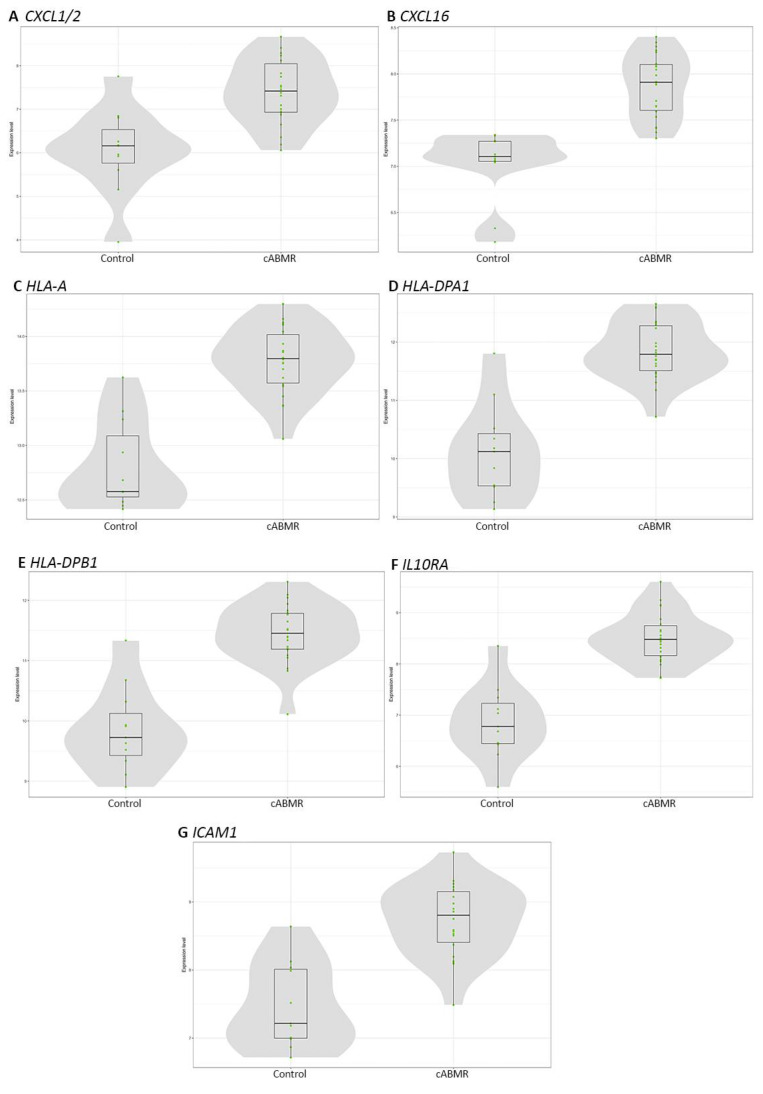
Box-plots of the most expressed genes in c-ABMR cases compared with the control group. *CXCL1/2* (**A**) and *CXCL16* (**B**) were among the genes most significantly expressed in c-ABMR, and they belonged to the CXCL family, a group of genes included in the chemokine signaling pathway. Similarly, *HLA-A* (**C**), *HLA-DPA1* (**D**), and *HLA-DPB1* (**E**) were part of the HLA group of genes with the most significant expression in c-ABMR. They were enlisted in several pathways including the adaptative immune system, the cell–ECM interaction, the MHC Class I Antigen Presentation, and the MHC Class II Antigen Presentation pathways. In addition, *IL10RA* (**F**) and *ICAM1* (**G**) were also among the most significantly expressed genes in c-ABMR, and were included in the cytokine signaling and T-reg differentiation in the former, and in the adaptive immune system, cell–ECM interaction, cytotoxicity, lymphocyte trafficking, NF-kappa B signaling, TNF family signaling, and type II interferon signaling in the latter. The grey areas represent the estimated genes’ expression distribution, while the green dots represent sample log2 expression for each gene.

The other most significantly overexpressed genes in c-ABMR belonged to the CXCL (*CXCL1/2, CXCL16*) and the HLA families (*HLA-A, HLA-DPA1, and HLA-DPB1*), the former involved in the chemokine signaling pathway, the latter in several pathways including the adaptative immune system, the cell–ECM interaction, the MHC Class I, and the MHC Class II antigen presentation pathways. In addition to these groups, *IL10RA* and *ICAM1* also were among the most significantly differentially expressed genes in c-ABMR (Figure 3). 

Based on the percentage of involved genes, the most altered pathways were MHC Class II antigen presentation (11/14 genes; 78.6%) and Type II interferon signaling (28/44 genes; 63.6%) (Table 6). 

**Figure 4:**
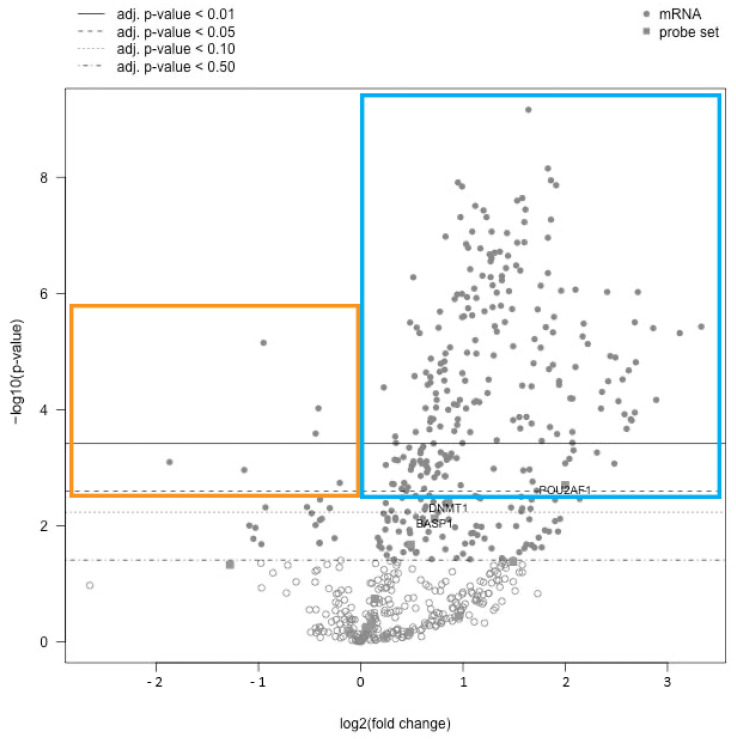
Gene expression profile. Volcano plot representing the gene expression profile of c-ABMR compared with the control group. The *x*-axis represents the fold change (log2) and the *y*-axis the gene’s *p*-value (−log10). Horizontal lines indicate adjusted *p*-value threshold. For our study, we considered statistically relevant the genes with a *p*-value < 0.05 (dashed line, second from top) or <0.01 (continuous line, first from top). Two hundred and twenty-one genes presented a statistically significant positive fold change and were therefore more expressed in the c-ABMR group (blue box), 170 of them with a *p*-value < 0.01 and 51 with a *p*-value < 0.05. Six genes presenting a statistically significant negative fold change were related to the control group (orange box).

Conversely, six genes were significantly more expressed in the control group, three of them with a *p*-value < 0.01 (*DEFB1, CHCHD10,* and *IGF1R*) and the remaining three with a *p*-value < 0.05 (*COL1A1, SLC4A1,* and *RAF1*) (Figure 4).

In agreement with the clinical and pathological findings, we did not observe any expression of RNAs related with viral infections. Expression signatures consistent with a more conspicuous inflammatory infiltrate were detected in c-ABMR and mast cells, exhausted CD8, and NK cells were the most represented inflammatory cells.

### 3.5. Gene Expression Profiling: C4d Positive versus C4d Negative c-ABMR Cases

We compared gene expression profiles of C4d-positive and negative cases within the c-ABMR group revealing no statistically significant differences (Figure S4), including the Cav-1 gene. This finding is consistent with the common diagnosis of c-ABMR of these samples and, more importantly, it backs up the role of Cav-1 IHC as a reliable surrogate marker of c-ABMR irrespective of C4d expression.

**Figure 5:**
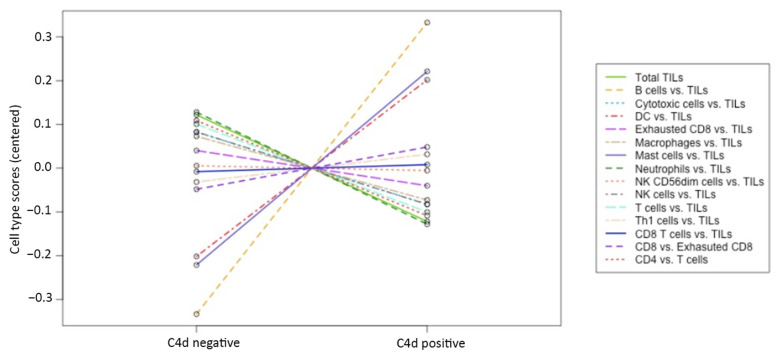
Inflammatory response. The plot represents the expression of gene signatures related to specific immune cell types comparing C4d positive versus C4d negative c-ABMR cases. Genes associated with B cell lymphocytes and mast-cells were more expressed in C4d positive cases, while genes related to T cell lymphocytes and NK cells were more expressed in C4d negative cases.

Nevertheless, we observed some differences between C4d-positive and negative cases concerning the gene-expression profile of the inflammatory infiltrate: signatures related to lymphocytes B and mast-cells were more represented in C4d-positive cases, while T lymphocytes and NK cells were predominantly expressed in C4d-negative cases. In the former group, *TNFRSF17*, *FAM30A*, *MS4A1* were the most expressed genes related to the B-cell response, while in the latter, *XCL1/2* emerged as the most expressed NK-related gene (Figure 5). 

Based on this finding, we decided to assess the IHC phenotype of the inflammatory infiltrate (B cell to T and NK cell ratio) to confirm the gene-expression profiling result. Although we could not evaluate all cases because of sample depletion, we identified a significant difference (*p* < 0.01) between C4d-positive and negative cases (a higher ratio was observed in C4d-positive cases compared with C4d-negative), thus confirming the molecular analysis result (Figure S5).

### 3.6. Gene Expression Profiling: Analysis of Caveolin-1 Expression and Significance in c-ABMR

**Figure 6:**
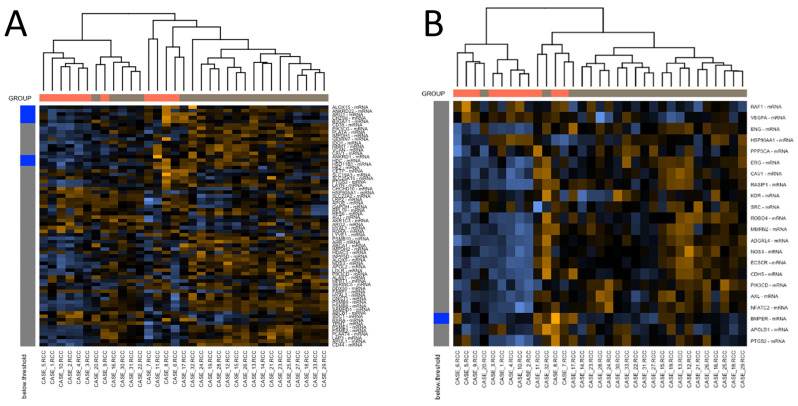
Heatmaps representation of the normalized genes included in the angiogenesis (**A**) and metabolism (**B**) pathway. In the angiogenesis pathway, *ROBO4, PIK3CD, ENG, RASIP1, NFATC2, CDH5, NOS3, ADGRL4, AXL, ECSCR* (*p* < 0.01) *MMRN2*, and Cav-1 (*p* < 0.50) were the genes significantly more expressed in c-ABMR cases. In the metabolism pathway, the significantly more expressed genes in the c-ABMR group were *APOL1, INPP5D, PSMB9, PSMB8, SAMHD1, ALOX5, PSME1, IDO1, PIK3CD, PSMB10, ABCA1, GNG11, PLA1A, PSME2, APOL2, NOS3, PLAAT4, AHR* (*p* < 0.01), *HYAL2, CD44, LAP3,* and Cav-1 (*p* < 0.05). The rows represent the normalized genes, the columns the biopsy samples. Within the cells, orange intensity is proportioned to increasing expression of the corresponding gene’s mRNA, while blue intensity is related to lower expression levels. On the left side of the heatmap, genes’ expressions below the detection threshold are represented by blue boxes, while gray boxes represent the ones above the detection threshold. Above the heatmap, orange boxes represent the control group cases, while gray boxes the c-ABMR cases. The dendrogram represents levels of interdependence among cases.

Considering that Cav-1 is involved in pathways related to angiogenesis and cellular metabolism, we decided to investigate the expression of other genes included within these pathways. In addition to Cav-1, the angiogenesis pathway presented 11 out of 22 more expressed genes in c-ABMR samples (*ROBO4, PIK3CD, ENG, RASIP1, NFATC2, CDH5, NOS3, ADGRL4, AXL, ECSCR* and *MMRN2*), ten of them with a *p*-value < 0.010. The metabolic pathway presented 21 out of 68 overexpressed genes (*APOL1, INPP5D, PSMB9, PSMB8, SAMHD1, ALOX5, PSME1, IDO1, PIK3CD, PSMB10, ABCA1, GNG11, PLA1A, PSME2, APOL2, NOS3, PLAAT4, AHR, HYAL2, CD44,* and *LAP3*), eighteen of them with a *p*-value < 0.01 (Figure 6). 

**Figure 7:**
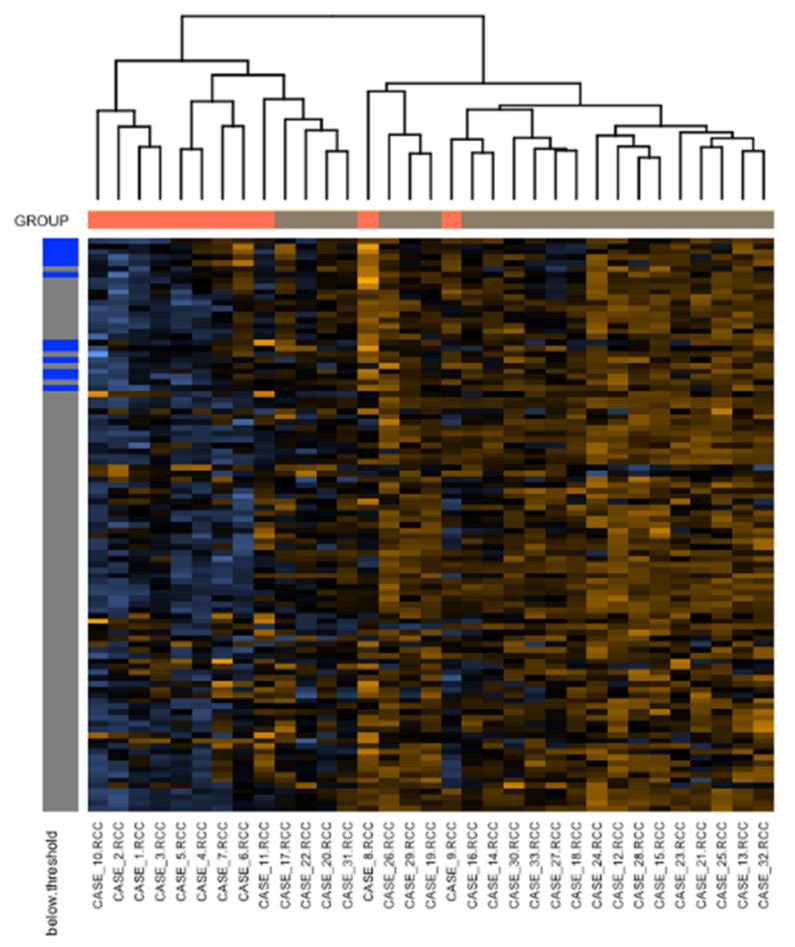
Heatmap representing the normalized genes expression of the cell–ECM interaction pathway. CD4, HLA-A, ITGB2, HLA-E, HLA-DRA, HLA-DPB1, HLA-DPA1, CTSS, PECAM1, HLA-F, HLA-DMB, HLA-DMA, ITGA4, HLA-B, ICAM1, VWF, ARHGDIB, ITGAX, VCAM1, PTPRC, MCAM, ICAM2, EMP3, CD40, TGFB1, HLA-DQB1, PSEN1, CDH5, MMP9, TIMP1, CD34, THBS1, HLA-DRB1, HLA-DRB3 (*p*-value < 0.01), CD44, MMP14, COL4A1, and CASP3 (*p*-value < 0.05) genes were significantly more expressed. The rows represent the normalized genes (individual genes’ names are not represented here due to space representation issues), the columns the biopsy samples. Within the cells, orange intensity is proportioned to increasing expression of the corresponding gene’s mRNA, while blue intensity is related to lower expression levels. On the left side of the heatmap, genes’ expressions below the detection threshold are represented by blue boxes, while gray boxes represent the ones above the detection threshold. Above the heatmap, orange boxes represent the control group cases, while gray boxes the c-ABMR cases. The dendrogram represents levels of interdependence among cases.

Furthermore, we detailed the cell–ECM interaction pathway, demonstrating that in c-ABMR, thirty-four genes were significantly more expressed (*CD4, HLA-A, ITGB2, HLA-E, HLA-DRA, HLA-DPB1, HLA-DPA1, CTSS, PECAM1, HLA-F, HLA-DMB, HLA-DMA, ITGA4, HLA-B, ICAM1, VWF, ARHGDIB, ITGAX, VCAM1, PTPRC, MCAM, ICAM2, EMP3, CD40, TGFB1, HLA-DQB1, PSEN1, CDH5, MMP9, TIMP1, CD34, THBS1, HLA-DRB1,* and *HLA-DRB3*), with a *p*-value < 0.01, and four with a *p*-value < 0.05 (Figure 7). 

Together with Cav-1, this latter pathway presented additional genes of potential clinical interest, such as *CD44, TGFB1, ICAM-1,* and *VCAM-1*. These four genes have been found to be related to focal and segmental glomerulosclerosis (FSGS), another relevant cause of long-term graft failure mediated by progressive fibrosis [37,38,39,40,41,42].

## 4. Discussion

c-ABMR represents a significant cause of graft failure after kidney transplantation. Pathological diagnosis of this entity is crucial to tailor patients’ clinical management, but it may prove challenging. We show that Cav-1 constitutes a reliable marker of c-ABMR, and its evaluation by IHC represents an accurate tool to assess its expression. We also provide evidence corroborating the value of nCounter^®^ Human Organ Transplant Panel for exploring the molecular landscape of c-ABMR. The characterization of c-ABMR-relevant molecular pathways provided here could help introduce novel potential diagnostic and therapeutic approaches for c-ABMR.

IHC assessment of linear C4d staining has long been considered a diagnostic hallmark of antibody-mediated rejection since its first demonstration in humoral rejection biopsies [11,12], and provides evidence of classical complement pathway activation, secondary to current/recent DSA interaction with graft endothelium. However, a complement-independent humoral immune response may be activated as well, and a significant number of antibody-mediated rejection cases are thought to be mediated by antibody-dependent cellular cytotoxicity (mostly by NK cell recruitment), consequently resulting C4d-negative [23]. Moreover, C4d-positivity also occurs in cases with no evidence of rejection [30,43,44,45]. For these reasons, C4d cannot be used as a surrogate marker of c-ABMR.

Cav-1 is the main scaffolding protein of caveolae: within the kidney, it is constitutively expressed by arterial smooth muscle cells, podocytes, mesangial cells, but not by endothelial cells [35,46,47,48]. Conversely, injured endothelial cells significantly express Cav-1 [28,29,30,31,32,33,34,35,49] and its expression has been found to be associated with arterial stiffness, allograft fibrosis and failure [33,50,51].

Although Cav-1 and C4d display a similar IHC staining pattern (i.e., linear peritubular and/or glomerular capillary positivity) they provide qualitatively different information: Cav-1 is a good marker of endothelial damage, showing immunoreactivity regardless of complement-activation and hence potentially supporting the c-ABMR diagnosis even in C4d negative cases.

Based on this evidence, we comprehensively investigated the correlation between Cav-1 expression and c-ABMR. Cav-1 staining was present in all c-ABMR cases of the present series, mainly showing a diffuse positivity both in peritubular and glomerular capillary endothelial cells with a slightly higher expression in the former vessels. Moreover, Cav-1 expression was independent of C4d status and no IHC expression was observed in endothelial cells of control cases supporting its diagnostic significance. In addition to this evidence, our exploratory IHC analysis on a small series of active ABMR and c-ABMR with other superimposed injury (e.g., mixed c-ABMR and TCMR, mixed c-ABMR and IgA nephropathy) suggested that Cav-1 could properly identify features of antibody-mediated injury even in these conditions. Notably, our results are in agreement with data reported in the literature, describing Cav-1 expression in cases with chronic rejection-induced TG [28] and antibody-mediated changes [30,32], further supporting its pathogenetic role in c-ABMR. In particular, Nakada et al. [30] explored Cav-1 immunohistochemical phenotype in a series of ninety-eight kidney transplant patients. They confirmed its expression in peritubular capillaries and its association with transplant glomerulopathy, and eventually correlated its positivity with an increased risk of graft failure. More recently, Teixeira et al. [32] assessed the expression of Caveolin-1 and other endothelial markers, such as von Willebrand factor and T-cadherin, in a series of cases with antibody-mediated changes and interstitial fibrosis and/or tubular atrophy, including C4d and C4d negative cases. They correlated Cav-1 expression with microvascular inflammation (*p* = 0.029) and antibody-mediated rejection (*p* = 0.016) and in particular chronic antibody-mediated rejection (*p* = 0.049). In agreement with our study, they did not observe any difference comparing C4d positive and C4d negative cases (*p* = 0.170). To further establish Cav-1 diagnostic reliability in c-ABMR we deemed it necessary to also show that Cav-1 gene expression discriminates c-ABMR from control samples. Molecular profiling employing the nCounter^®^ Human Organ Transplant Panel showed that: (i) the c-ABMR series and the control samples harbored clearly distinct gene expression profiles; (ii) no significant differences existed in terms of gene expression between C4d positive and C4d negative cases; (iii) Cav-1 gene expression was significantly higher in c-ABMR versus control series, independent of C4d status. Thus, gene expression confirmed the specific relevance of Cav-1 in c-ABMR and corroborated its significance as a surrogate marker of c-ABMR irrespective of C4d status.

To the best of our knowledge, this is the first study proving Cav-1 IHC expression as a reliable supporting diagnostic marker of antibody-mediated rejection through an integrated IHC and molecular analysis [16,52,53,54].

As such, a crucial issue is whether Cav-1 may be expressed in conditions other than antibody-mediated rejection. In our experience (unpublished data), Cav-1 is also expressed in transplant and native kidney biopsies showing features consistent with chronic/active thrombotic microangiopathy (TMA) and exhibiting a staining pattern similar to c-ABMR. Although this finding supports its reliability as a complement-independent endothelial-damage marker, this positivity also represents a limitation of Cav-1 in distinguishing de novo or relapsing TMA from true antibody-mediated rejection, which may sometimes present TMA morphological features. However, this distinction is possible based on the clinical setting, thus underlining the critical importance of clinic-pathological correlations in transplant-related conditions. Conversely, we did not observe any focal (Grade II) nor diffuse (Grade III) Cav-1 immunoreactivity in several kidney conditions (e.g., arteriosclerosis-related vascular injury, interstitial fibrosis, post-transplant membranous glomerulonephritis, acute pyelonephritis, acute tubular necrosis, diabetic nephropathy plus recurrent 2,8 DHA nephropathy, TCMR). Although based on a small series of cases, these findings suggest a sufficient specificity of Cav-1 for antibody-mediated injury, but further studies comprising a larger number of cases will be necessary to fully enable the use of Cav-1 in daily diagnostic practice.

Gene expression profiling provided additional valuable data concerning the c-ABMR transcriptomic landscape, thus supporting the efficacy of these tools for characterizing transplant-related conditions. A comparison between the c-ABMR cases and the control group revealed a significantly higher expression of genes related to humoral rejection in the first group. Among other relevant pathways, identification of early parenchymal fibrosis is of compelling interest to prevent allograft failure: genes involved in the cell–ECM interaction pathway (including fibrogenesis-relevant genes such as *CD44, TGFB1, ICAM-1, and VCAM-1*) were significantly overexpressed in c-ABMR cases compared with controls series, but we did not observe any significant correlation with global/segmental glomerular sclerosis.

Furthermore, although no significant differences were detected between C4d positive and C4d-negative cases, a trend of different expression for genes related to the type of immune cell infiltrate was observed: specifically, B-cell genes, *TNFRSF17* in particular, were more expressed in C4d-positive cases. *TNFRSF17*, encoding for the B-Cell Maturation Antigen (BCMA), is currently being actively investigated as a potential therapeutic target in hematological malignancies such as multiple myeloma [55,56,57,58]. According to our results, anti-BCMA treatments could be considered as an innovative target for tackling B cell-driven antibody-mediated rejection. Conversely, C4d negative cases turned out to overexpress NK cells related-genes, further supporting their role in C4d-negative antibody-mediated rejection. The most significantly higher expressed gene related to NK cells in C4d negative cases was *XCL1/2* (also known as lymphotactin), a cytokine belonging to the C-family chemokines and secreted by NK cells to recruit and activate CD8-positive dendritic cells. This interaction allows NK cells to support the survival and differentiation of CD8-positive T-cells into a cytotoxic phenotype [59,60]. In addition, the IHC analysis confirmed the molecular findings, showing a higher B cell to T and NK cell ratio in C4d-positive c-ABMR samples. Based on this evidence, further studies focused on the innate and adaptive cytotoxic response and the targeting of NK cells could be explored for new therapeutic strategies in C4d-negative antibody-mediated rejection cases.

Finally, some technical considerations are also worth mentioning. To date, most published studies about kidney graft molecular profiling were performed on a tissue sample different from the one submitted to histological evaluation [27]. This approach may lead to inconsistencies and misleading correlations between morphology and molecular analysis [61]. By employing FFPE-based gene expression analysis, we overcame this issue since it was possible to use the same specimen for both evaluations. Concerning the fixation protocol of our samples, in our experience the use of AFA solution for the fixation of kidney biopsies results in superior morphological preservation along with comparable IHC performances [62,63]; thus for many years our institution has preferred it to buffered formalin for routine diagnostics [64,65,66,67]. Nevertheless, AFA and buffered formalin include the same amount of formaldehyde, the component most likely compromising the quality of nucleic acids, and thus no negative effect on mRNA samples should be expected [68,69,70]. Accordingly, our mRNA samples showed no quality concerns.

Our study has some potential limitations, including its retrospective nature, the limited sample size, and the non-consecutive collection of samples. Considering the aims of the study, we deemed the strictness of the inclusion criteria to be more important than sample size or consecutive recruitment; accordingly, our case series was strictly selected to avoid possible confounders.

In conclusion, our data support the biological significance of Cav-1 in c-ABMR and the promising diagnostic role of its IHC, providing proof of its simultaneous mRNA and protein expression, and also backing up the reliability of the nCounter^®^ Human Organ Transplant Panel for detecting and investigating antibody-mediated rejection. We believe that Cav-1 IHC staining may represent a valuable tool, especially to enable the correct assessment of C4d-negative c-ABMR cases.

Future research directions should include the assessment of Cav-1 expression in active antibody-mediated rejection, in cases with initial c-ABMR-related lesions (i.e., cg1a transplant glomerulopathy, peritubular capillary basement membrane multilayering) which are still undetectable by light microscopy, and in larger series with multiple transplant-related differential diagnoses including overlapping and challenging cases.

## Figures and Tables

**Table 1:** Caveolin-1 grading system. Cav-1 grading was built similarly to the Banff C4d score. Each score must be performed separately for peritubular and glomerular capillaries: grade 0/negative was attributed to cases with no Cav-1 expression; grade I/minimal to cases with a <10% positivity in peritubular–glomerular capillaries; grade II/focal if the positivity was between 10% and 50%; grade III/diffuse if Cav-1 was >50% in peritubular–glomerular capillaries.

Cav-1 Expression	Percentage of Positive Peritubular Capillaries or Glomeruli	Score
Negative	0	Grade 0
Minimal	1—<10	Grade I
Focal	10—50	Grade II
Diffuse	>50	Grade III

**Table 2:** Clinicopathological characteristics of c-ABMR cases compared with the control group. CKD: chronic kidney disease; APKD: polycystic kidney disease; PTO: proteinuria; Crs: creatininemia; g: glomerulitis; ptc: peritubular capillaritis.

Characteristics		c-ABMR (*n* = 22)	Control Group (*n* = 11)	*p*-Value
Gender	Female	7	6	0.208
	Male	15	5	
Age at diagnosis	Median (interval)	54 (17–70)	49 (31–69)	0.902
Native disease	Idiopathic CKD	5	3	0.964
	Immune-mediated glomerulonephritis	5	3	
	APKD	4	1	
	Urinary tract malformation	4	1	
	Idiopathic chronic glomerular disease	2	1	
	Alport syndrome	1	1	
	Other	1	1	
Donor type	Deceased	17	11	0.086
	Living	5	0	
Transplanted kidney	Single	22	10	0.151
	Double	0	1	
Re-transplantation	No	19	11	0.199
	Yes	3	0	
Comorbidities	No	8	2	0.284
	Yes	14	9	
Comorbidities type	None	8	2	0.077
	Hypertension	6	2	
	Hypertension and metabolic disorders	1	3	
	Hypertension and other causes	1	3	
	Other	6	1	
Treatment	Single-immunosuppressant therapy	7	3	0.106
	Double-immunosuppressant therapy	9	8	
	Triple-immunosuppressant therapy	6	0	
DSA	Not applicable	0	11	-
	Class I	8	0	
	Class II	12	0	
	Both	2	0	
PTO (g/24 h)	Median (interval)	0.875 (0.2–8)	0.12 (0–0.46)	0.026
Crs (mg/dL)	Median (interval)	2.55 (1.25–4.6)	1.49 (0.71–3.67)	0.013
Focal segmental glomerulosclerosis (FSGS)	Not applicable	0	11	-
	No	12	0	
	Yes	10	0	
Active ABMR (g and/or ptc)	Not applicable	0	11	-
	No	8	0	
	Yes	14	0	
Glomerulitis score (g)	Not applicable	0	11	-
	0	11	0	
	1	6	0	
	2	4	0	
	3	1	0	
Peritubular capillaritis score (ptc)	Not applicable	0	11	-
	0	9	0	
	1	2	0	
	2	10	0	
	3	1	0	
Transplant glomerulopathy score (TG)	Not applicable	0	11	-
	0	0	0	
	1	3	0	
	2	6	0	
	3	13	0	
C4d score	Not applicable	0	11	-
	0	12	0	
	1	2	0	
	2	2	0	
	3	6	0	
Graft failure	No	19	11	0.199
	Yes	3	0	
Median follow-up	(25th–75th)	9.9 (6.4–13.3)	4.6 (1.9–5.6)	-
Median rejection time	(25th–75th)	5.6 (3.6–9.8)	Not applicable	-

**Table 3:** Clinicopathological data of C4d positive versus C4d negative cases. CKD: chronic kidney disease; APKD: polycystic kidney disease; PTO: proteinuria; Crs: creatininemia; g: glomerulitis; ptc: peritubular capillaritis.

		C4d Negative (*n* = 12)	C4d Positive (*n* = 10)	*p*-Value (*: Log Rank Test)
Gender	Female	5	2	0.277
	Male	7	8	
Age at diagnosis	Median (interval)	53 (18–70)	55 (17–69)	0.669
Native disease	Idiopathic CKD	3	2	0.247
	Immune-mediated glomerulonephritis	1	4	
	APKD	4	0	
	Urinary tract malformation	2	2	
	Idiopathic chronic glomerular disease	1	1	
	Alport syndrome	0	1	
	Other	1	0	
Donor type	Deceased	10	7	0.457
	Living	2	3	
Re-transplantation	No	9	10	0.089
	Yes	3	0	
Comorbidities	No	4	4	0.746
	Yes	8	6	
Comorbidities type	None	4	4	0.529
	Hypertension	4	2	
	Hypertension and metabolic disorders	0	1	
	Hypertension and other causes	0	1	
	Other	4	2	
Treatment	Single-immunosuppressant therapy	3	4	0.616
	Double-immunosuppressant therapy	6	3	
	Triple-immunosuppressant therapy	3	3	
DSA	Class I	6	2	0.146
	Class II	6	6	
	Both	0	2	
PTO (g/24 h)	Median (interval)	0.58 (0.2–8)	1.22 (0.5–5.91)	0.468
Crs (mg/dL)	Median (interval)	2.85 (1.25–3.4)	2.1 (1.6–4.6)	0.905
Focal segmental glomerulosclerosis (FSGS)	No	7	5	0.696
	Yes	5	5	
Active ABMR (g and/or ptc)	No	4	4	0.746
	Yes	8	6	
Glomerulitis score (g)	0	5	6	0.458
	1	4	2	
	2	3	1	
	3	0	1	
Peritubular capillaritis score (ptc)	0	4	5	0.340
	1	2	0	
	2	6	4	
	3	0	1	
Transplant glomerulopathy score (TG)	1	3	0	0.122
	2	4	2	
	3	5	8	
Graft failure	No	9	10	0.089
	Yes	3	0	
Median follow-up	(25th–75th)	7.4 (5.6–12.6)	10.4 (8.7–21.3)	0.061 *
Median rejection time	(25th–75th)	4.3 (2.2–6.1)	8.5 (5.6–20.4)	0.023 *

**Table 4:** Cav-1 IHC score results in c-ABMR and control samples.

			c-ABMR (*n* = 22)	Control Group (*n* = 11)	*p*-Value
Cav-1 peritubular capillaries expression score	Expression	Negative	0	11	<0.0001
		Positive	22	0	
	Score	Grade 0	0	11	-
		Grade I	0	0	
		Grade II	2	0	
		Grade III	20	0	
Cav-1 glomerular capillaries expression score	Expression	Negative	0	11	<0.0001
		Positive	22	0	
	Score	Grade 0	0	11	-
		Grade I	4	0	
		Grade II	11	0	
		Grade III	7	0	

**Table 5:** Cav-1 IHC score results in c-ABMR samples according to C4d status.

			C4d Negative (*n* = 12)	C4d Positive (*n* = 10)	*p*-Value
Cav-1 peritubular capillaries expression score	Expression	Negative	0	0	-
		Positive	12	10	
	Score	Grade I	0	0	0.176
		Grade II	2	0	
		Grade III	10	10	
Cav-1 glomerular capillaries expression score	Expression	Negative	0	0	-
		Positive	12	10	
	Score	Grade I	3	1	0.035
		Grade II	8	3	
		Grade III	1	6	

**Table 6:** Overexpressed genes (OGs) in c-ABMR compared with control samples according to nCounter^®^ Human Organ Transplant annotated pathways.

Annotated Pathways	Total Number of Genes Analyzed	Total Number of Overexpressed Genes (Percentage)	# OGs (*p* < 0.01)	# OGs (*p* < 0.05)
Adaptive Immune System	127	52 (40.9)	48	4
Angiogenesis	22	12 (54.5)	10	2
Apoptosis and Cell Cycle Regulation	52	23 (44.2)	17	6
Autophagy	17	3 (17.6)	1	2
B-cell Receptor Signaling	45	24 (53.3)	21	3
Cell–ECM Interaction	101	39 (38.6)	34	5
Chemokine Signaling	57	19 (33.3)	16	3
Complement System	31	12 (38.7)	8	4
Cytokine Signaling	98	15 (15.3)	13	2
Cytosolic DNA Sensing	19	5 (26.3)	3	2
Cytotoxicity	54	21 (38.9)	19	2
Epigenetics and Transcription	16	1 (6.2)	0	1
Hematopoiesis	204	62 (30.4)	43	19
Inflammasomes	11	4 (36.4)	2	2
Innate Immune System	165	59 (35.7)	46	13
Lymphocyte Trafficking	21	11 (52.4)	10	1
MAPK	63	15 (23.8)	7	8
Metabolism	68	22 (32.3)	18	4
MHC Class I Antigen Presentation	33	19 (57.5)	18	1
MHC Class II Antigen Presentation	14	11 (78.6)	11	0
mTOR	14	3 (21.4)	1	2
NF-kappa B Signaling	56	20 (35.7)	17	3
NLR Signaling	54	18 (33.3)	14	4
Oxidative Stress	62	15 (24.2)	10	5
T-cell Checkpoint Signaling	28	4 (14.3)	4	0
T-cell Receptor Signaling	66	28 (42.4)	25	3
TGF-beta Signaling	30	6 (20.0)	4	2
Th1 Differentiation	16	6 (37.5)	4	2
Th17 Differentiation	39	8 (20.5)	5	3
Th17 Mediated Biology	39	9 (23.1)	7	2
Th2 Differentiation	17	5 (29.4)	4	1
Tissue Homeostasis	39	8 (20.5)	3	5
TNF Family Signaling	61	19 (31.1)	13	6
Toll-like Receptor Signaling	70	21 (30.0)	17	4
Treg Differentiation	13	5 (38.5)	3	2
Type I Interferon Signaling	39	19 (48.7)	16	3
Type II Interferon Signaling	44	28 (63.6)	26	2
Viral Detection	4	0 (0)	0	0

## Data Availability

Data supporting the findings of the present study are not publicly available due to privacy/ethical restrictions, but can be obtained from the corresponding author upon reasonable request.

## Supplementary Materials

The following are available online at https://www.mdpi.com/article/10.3390/biomedicines9101318/s1, Figure S1. Follow-up time analysis overall population, Figure S2. Follow-up time analysis according to C4d status, Figure S3. Cav-1 immunohistochemical expression in additional transplant kidney diseases, Figure S4. Gene expression profile according to C4d status, Figure S5. B cell to T and NK cell ratio box plot, Methods M1, Table S1. Details of Cav-1 IHC expression in the exploratory series of additional kidney pathological conditions.
