# Supplementary material for: Caveolin-1 in Kidney Chronic Antibody-Mediated Rejection: An Integrated Immunohistochemical and Transcriptomic Analysis Based on the Banff Human Organ Transplant (B-HOT) Gene Panel
Source: Biomedicines. 2021 Sep 26;9(10):1318. doi: 10.3390/biomedicines9101318 (PMC8533527; doi:10.3390/biomedicines9101318)
Supplement: Supplementary file 1 [file biomedicines-09-01318-s001.zip › biomedicines-1363592-supplementary.pdf]

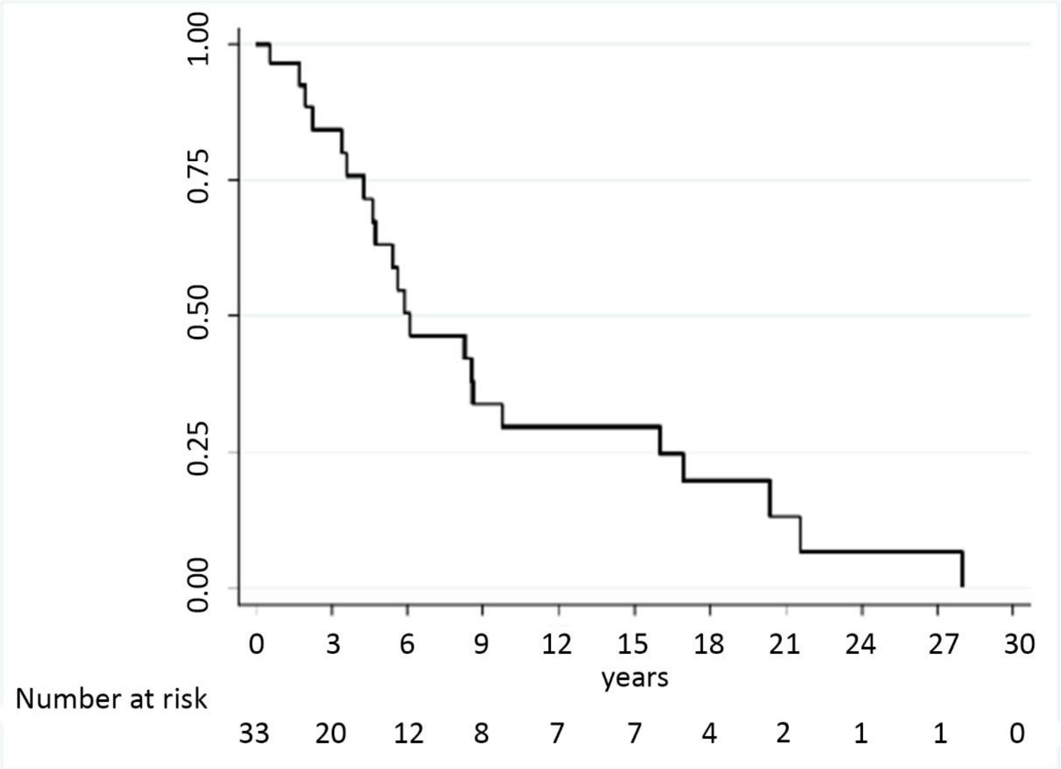

Figure S1 Follow-up time analysis. The Kaplan-Meyer method was applied on the study's population, considering c-ABMR and the control group.

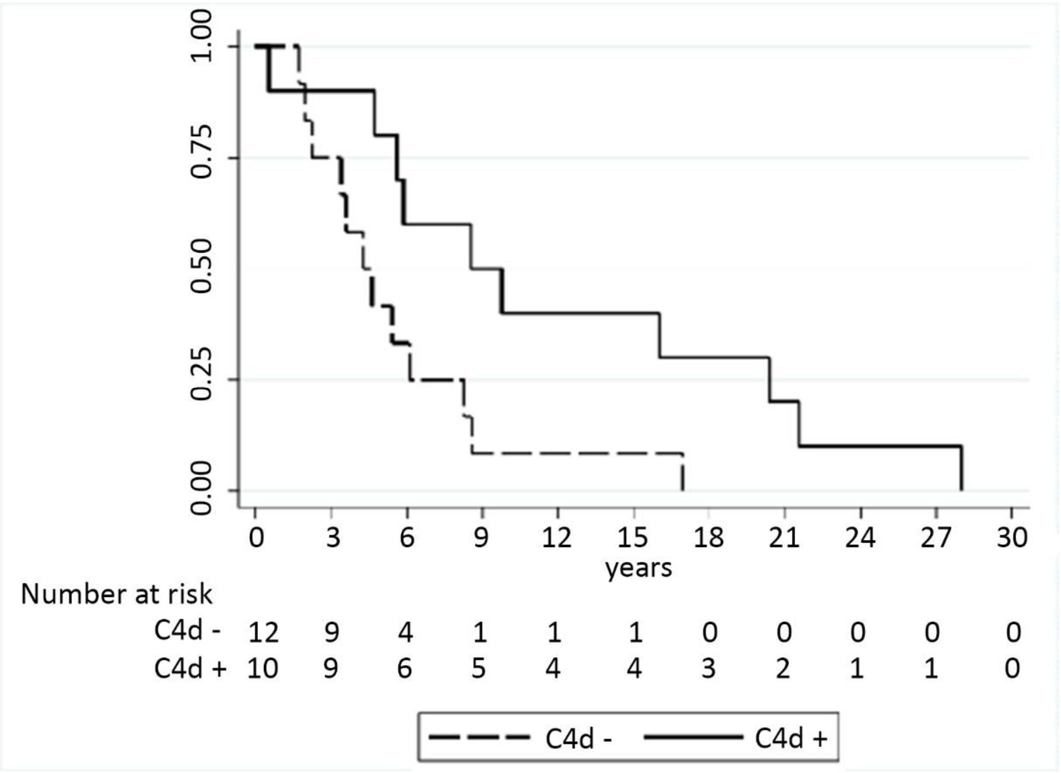

Figure S2 Follow-up time analysis. The Kaplan-Meier method was performed to represent the median rejection time comparing the C4d positive (10 cases) and the C4d negative (12 cases) group (p=0.023).

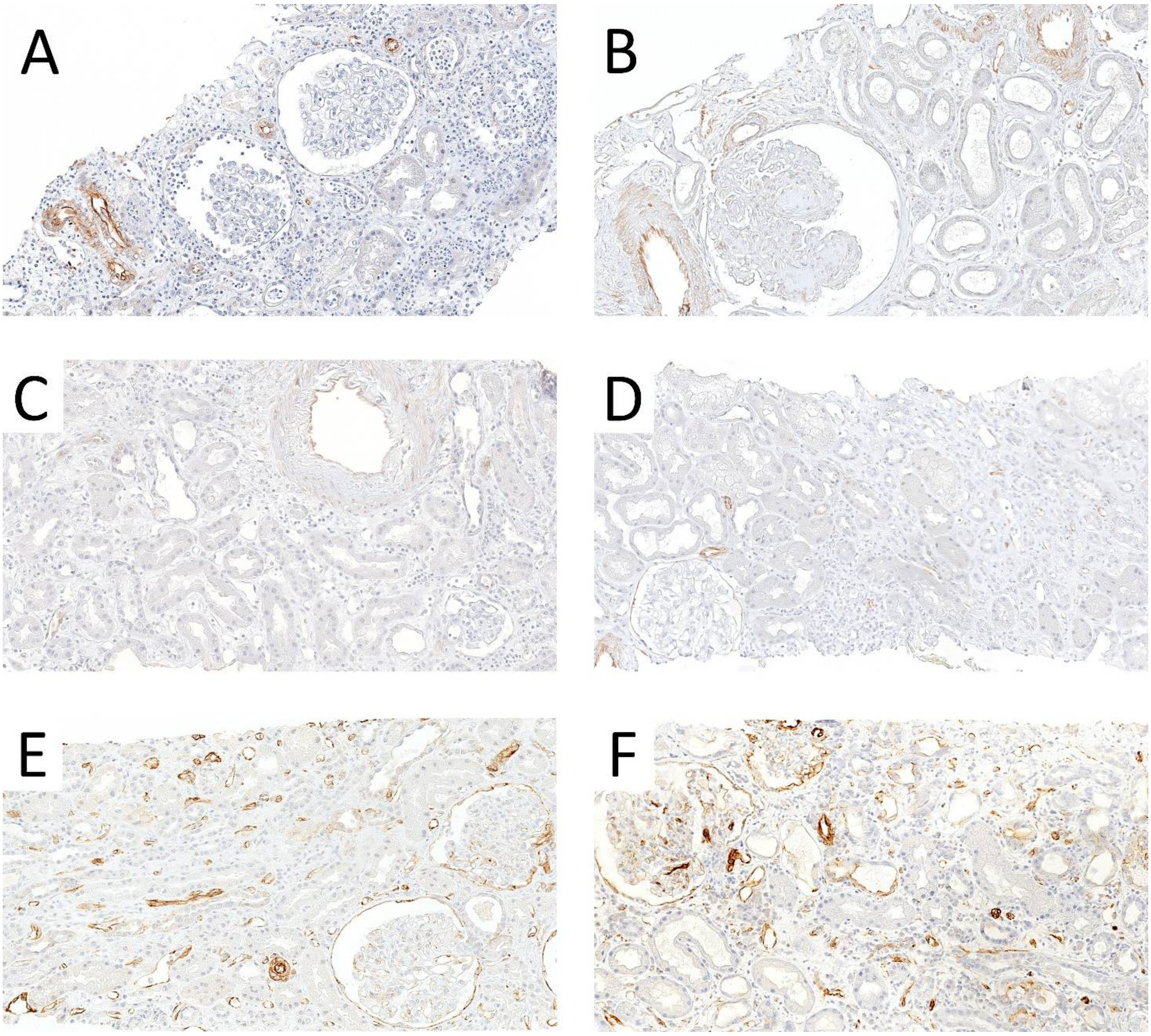

Figure S3 Cav-1 immunohistochemical expression in additional transplant kidney diseases. In diseases with no histopathological features of an antibody mediated injury, Cav-1 resulted completely negative (Grade 0) in glomerular and peritubular capillaries (A: acute pyelonephritis) or with a minimal expression (Grade 1) in peritubular capillaries only (B: diabetic nephropathy plus recurrent 2,8 DHA nephropathy; C: acute tubular necrosis; D: pure TCMR). Conversely, samples that presented an antibody mediated injury were Cav-1 focal or diffuse positive (Grade 2-3) (E: active ABMR; F: c-ABMR with superimposed IgA nephropathy). Original magnification 200x.

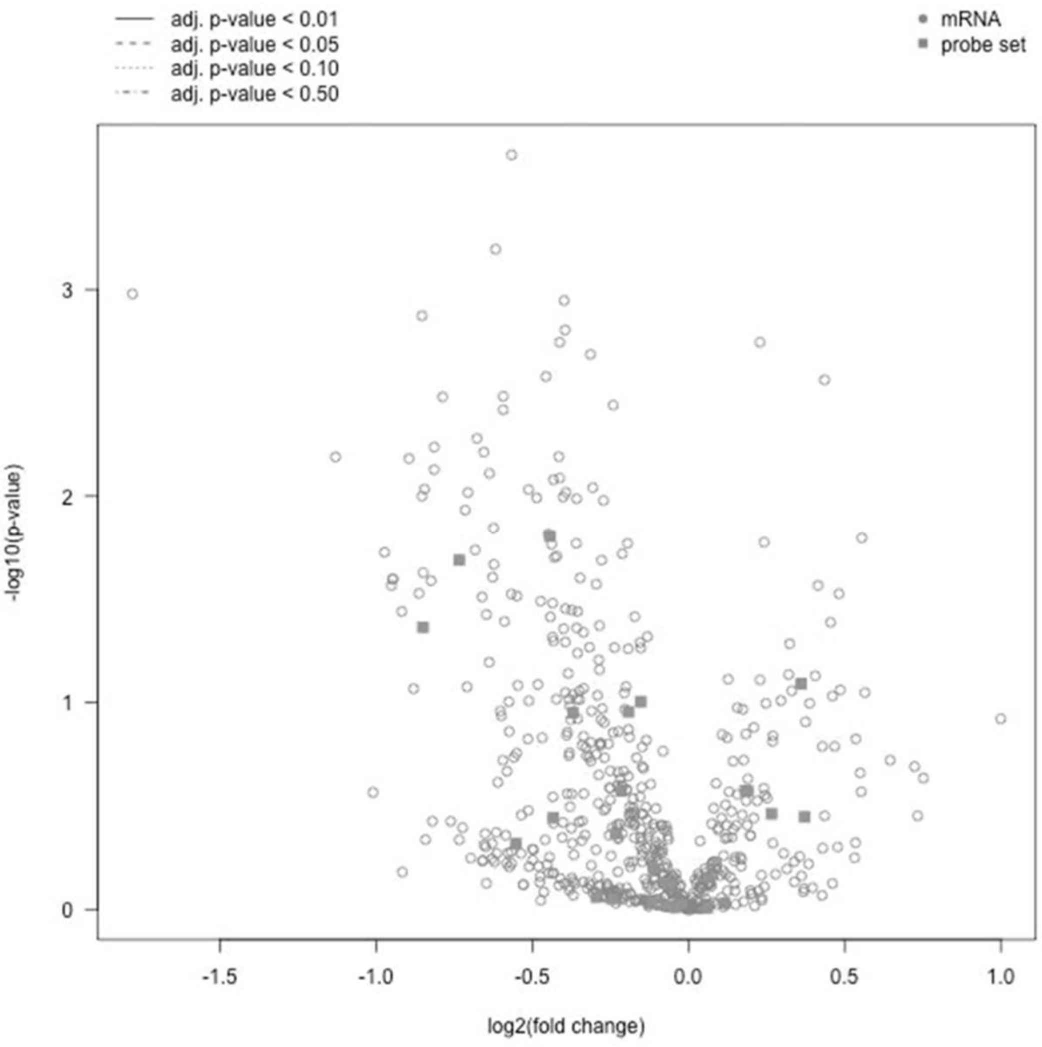

Figure S4 Gene expression profile. Volcano plot representing the gene expression profile of C4d positive compared to C4d negative cases. In this plot, no horizontal lines P-value threshold are represented since no genes presented a statistical relevant fold change (all genes' expression presented a P-value >0.50).

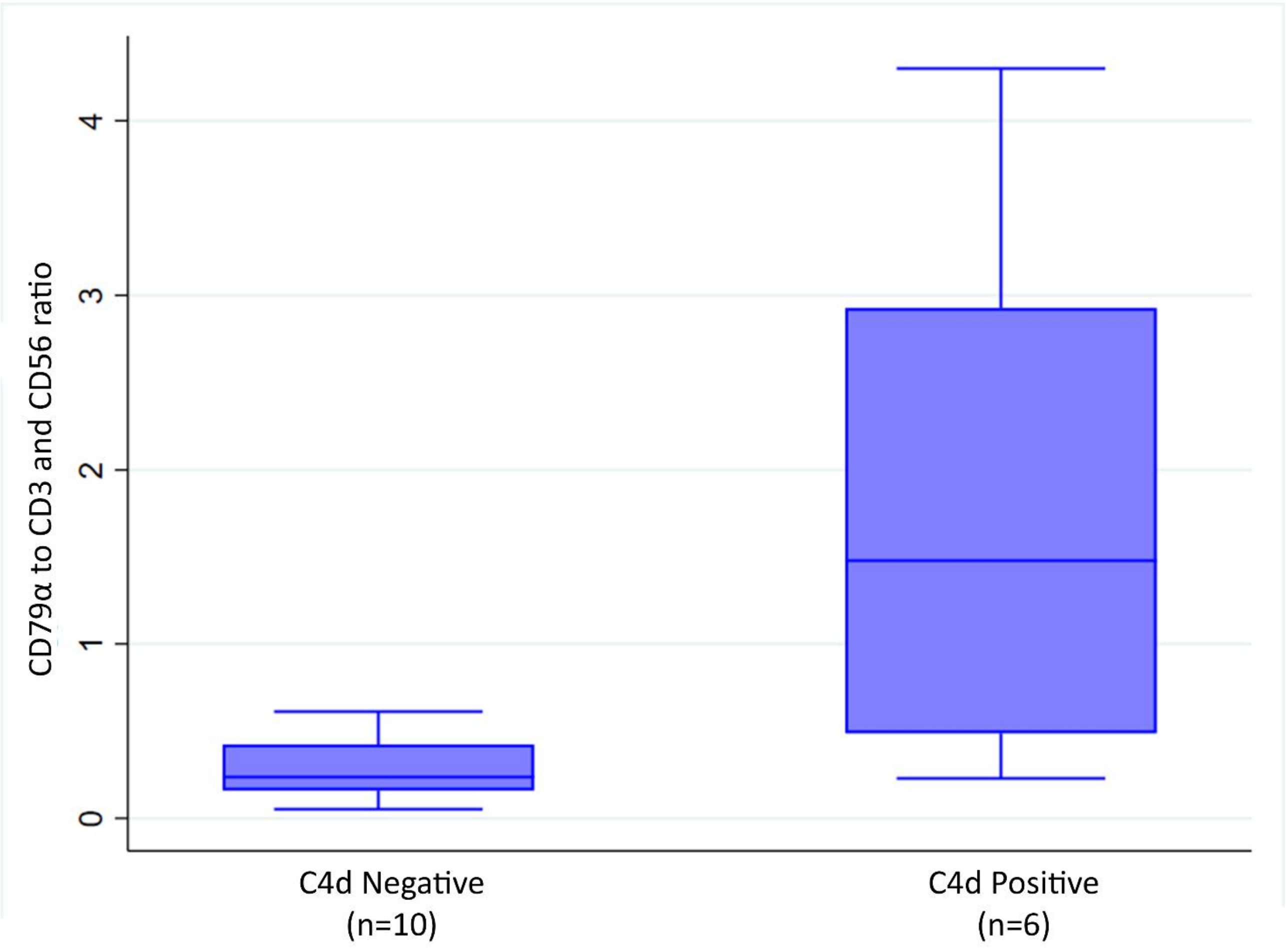

Figure S5 Box plot of B cell to T and NK cell ratio in C4d-negative versus C4d-positive c-ABMR samples based on IHC evaluation.

	Cav-1 expression score							
	Peritubular capillaries				Glomerular capillaries			
	Grade 0	Grade 1	Grade 2	Grade 3	Grade 0	Grade 1	Grade 2	Grade 3
**Arteriosclerosis-related vascular injury (n=2)**	2	0	0	0	2	0	0	0
**Interstitial fibrosis (n=1)**	1	0	0	0	1	0	0	0
**Post-transplant membranous glomerulonephritis (n=2)**	2	0	0	0	2	0	0	0
**Acute pyelonephritis (n=1)**	1	0	0	0	1	0	0	0
**Acute tubular necrosis (n=1)**	0	1	0	0	1	0	0	0
**Diabetic nephropathy plus recurrent 2,8 DHA nephropathy (n=1)**	0	1	0	0	1	0	0	0
**T-Cell Mediated Rejection (TCMR) (n=5)**	1	4	0	0	5	0	0	0
**Active antibody-mediated rejection (n=10)**	0	0	0	10	1	3	4	2
**Mixed c-ABMR/TCMR (n=1)**	0	0	0	1	0	0	0	1
**Mixed c-ABMR/IgA nephropathy (n=4)**	0	0	0	4	0	0	4	0

Table S1 Details of Cav-1 IHC expression in the exploratory series of additional kidney pathological conditions.

Methods M1 All samples were fixed with alcohol-formalin-acetic acid (AFA) and paraffin-embedded (PE). Two-μm sections were then cut, mounted onto adhesive slides, and stained with hematoxylin-eosin, periodic acid-Schiff, Masson's trichrome, phosphotungstic acid hematoxylin, acid fuchsin orange G, and Jones' methenamine silver stain. In all cases, deposition of C4d was determined by IHC (clone SP91; Ventana Medical Systems, Arizona, USA).

In some cases, tissue specimens were also routinely analyzed with direct immunofluorescence (DIF) for diagnostic purposes. For DIF, fresh-frozen tissue samples were cut at three μm-thick in a cryostat, fixed for 10 minutes in cold acetone, and then stained with a panel of FITC-conjugated antibodies (IgA, IgM, IgG, C3, C4, C1q, and fibrinogen; New Scientific Company, Lombardy, Italy).

All immunohistochemical stainings were performed with the automated immunostainer BenchMark XT AutoStainer® (Ventana Medical Systems, Arizona, USA).

All the procedures mentioned in the manuscript were performed according to manufacturer's protocols and instructions.
